# Domes and cones: Adhesion-induced fission of membranes by ESCRT proteins

**DOI:** 10.1371/journal.pcbi.1006422

**Published:** 2018-08-21

**Authors:** Jaime Agudo-Canalejo, Reinhard Lipowsky

**Affiliations:** 1 Theory & Bio-Systems Department, Max Planck Institute of Colloids and Interfaces, Potsdam, Germany; 2 Rudolf Peierls Centre for Theoretical Physics, University of Oxford, Oxford, United Kingdom; 3 Department of Chemistry, The Pennsylvania State University, University Park, Pennsylvania, United States of America; University of Virginia, UNITED STATES

## Abstract

ESCRT proteins participate in the fission step of exocytic membrane budding, by assisting in the closure and scission of the membrane neck that connects the nascent bud to the plasma membrane. However, the precise mechanism by which the proteins achieve this so-called reverse-topology membrane scission remains to be elucidated. One mechanism is described by the *dome model*, which postulates that ESCRT-III proteins assemble in the shape of a hemispherical dome at the location of the neck, and guide the closure of this neck *via* membrane–protein adhesion. A different mechanism is described by the *flattening cone model*, in which the ESCRT-III complex first assembles at the neck in the shape of a cone, which then flattens leading to neck closure. Here, we use the theoretical framework of curvature elasticity and membrane–protein adhesion to quantitatively describe and compare both mechanisms. This comparison shows that the minimal adhesive strength of the membrane–protein interactions required for scission is much lower for cones than for domes, and that the geometric constraints on the shape of the assembly required to induce scission are more stringent for domes than for cones. Finally, we compute for the first time the adhesion-induced constriction forces exerted by the ESCRT assemblies onto the membrane necks. These forces are higher for cones and of the order of 100 pN.

## Author summary

Membrane fission is a crucial step in many biological processes ranging from cell division to viral budding. During fission, the membrane forms a narrow neck that is subsequently cleaved by proteins. ESCRT proteins initiate this process by forming supramolecular assemblies that adhere to the cytosolic face of the membranes. However, how these assemblies achieve the closure and scission of membrane necks is still a matter of controversy. Here, we elucidate and compare the two most prominent mechanisms that have been proposed for ESCRT-induced membrane fission. In both mechanisms, the ESCRT molecules form assemblies that adhere to the open neck of the membrane bud. In the first mechanism, these assemblies grow in a dome-like shape, whereas in the second mechanism the assemblies take a cone-like shape and flatten as they grow. Our computational study shows that the minimal strength of membrane–protein adhesion necessary for cone-induced fission is lower than for dome-induced fission, and that cones generate higher constriction forces of the order of 100 pN onto the membrane necks.

## Introduction

Membrane remodelling through fission is a fundamental process in all living cells. It is a key step in various cellular tasks such as cell division, organelle biogenesis, endosomal sorting, membrane trafficking, viral budding, or endocytosis. [1–10] Because of its importance, the *in vivo* process is tightly controlled by proteins that assemble at the neck of the nascent membrane bud and cleave it. Two different kinds of fission events can be distinguished, however, which involve fundamentally different protein machinery: fission of buds that form towards the cytosol (endocytic), and fission of buds that form away from the cytosol (exocytic). In the first case, the cellular machinery (which ‘lives’ in the cytosol) can easily assemble at the neck and cleave it, for example by directly exerting constriction forces on this neck as in the case of dynamin proteins, [9, 10] while remaining within the cytosol. In the second case, however, how a cytosolic protein can assemble at the neck and induce its scission, while remaining at the cytosolic side of the membrane, becomes a tricky question. The latter process is sometimes referred to as reverse-topology membrane scission. [1]

The endosomal sorting complex required for transport (ESCRT) proteins are responsible for carrying out this process, although the precise mechanism by which they do it is unknown. The ESCRT machinery is rather complex, involving at least the four ESCRT complexes known as ESCRT-0, ESCRT-I, ESCRT-II, ESCRT-III, as well as the associated ATPase protein Vps4. [1–4] It is believed that ESCRT-III and Vps4 are the key actors in membrane fission, with recent studies showing that Vps4 and ESCRT-III cooperate in a highly dynamic fashion throughout the whole process, [11, 12] while the other ESCRTs act upstream to recruit them. [13] ESCRT-III is composed of four subunits known as Vps20, Snf7, Vps2 and Vps24 in yeast nomenclature (respectively known as CHMP6, CHMP4, CHMP2 and CHMP3 in animal cells), which are monomers in solution that can bind to the membrane. Snf7 is the main component of ESCRT-III, and has been shown to assemble into long membrane-bound protein spirals *in vitro*. [14] Vps20 acts to nucleate Snf7, while Vps2 and Vps24 act to block polymerization by binding along the Snf7 filaments. [11] In addition to nucleation and growth, the dynamics of ESCRT-III includes the continuous addition and removal of subunits during the lifetime of the protein complex.

**Fig 1:**
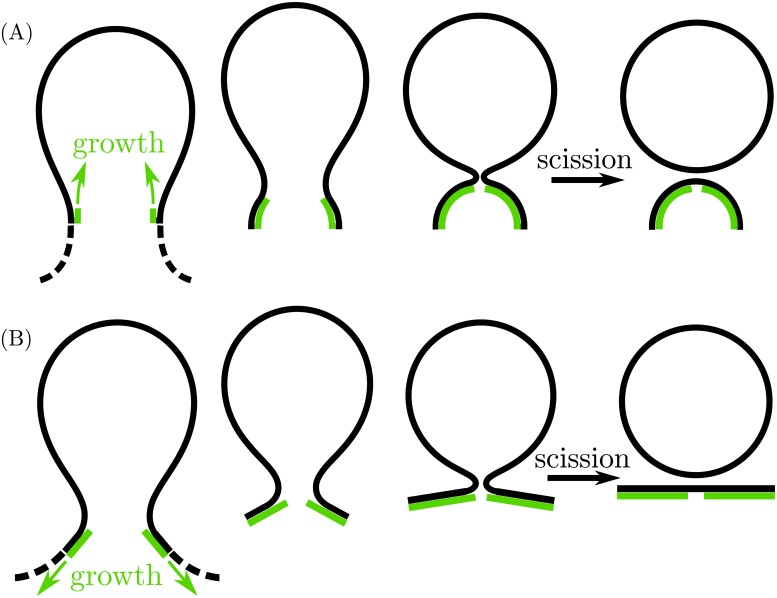
Proposed mechanisms for adhesion-induced fission consisting of neck closure and scission by dome-shaped and cone-shaped ESCRT complexes. (A) In the dome model, the ESCRT-III complex (green) first assembles at the neck of a nascent bud, and then grows *towards* the bud, forming a hemispherical dome. Because of membrane–protein adhesion, the membrane (black) follows the growth of the dome until the diameter of the neck becomes comparable to the membrane thickness, and the neck can undergo scission. (B) In the flattening cone model, the ESCRT-III complex first assembles at the neck of the nascent bud, and then grows *away from* the bud in the shape of a cone. As the truncated cone grows beyond a critical size, it flattens, leading to closure and scission of the membrane neck. All cartoons represent axisymmetric geometries.

How ESCRT-III manages to close and scission the membrane neck is a matter of debate. One mechanism is described by the *dome model*, [1, 15, 16] which proposes that the ESCRT-III complex assembles at the neck of the membrane bud, and grows towards the bud forming consecutively narrower spiral rings, with the overall shape resembling a dome. If the interactions between the protein dome and the membrane are sufficiently attractive, the membrane will follow the growth of the dome until the neck of the bud closes and scission can occur, see Fig 1A. [15] The dome model was in part inspired by the observation of dome-like structures assembled *in vitro* from ESCRT components in Ref [17], although it should be noted that the latter structures were composed of only two different ESCRT-III subunits (CHMP2AΔC-CHMP3ΔC) subject to C-terminal mutations, and therefore are not directly comparable to *in vivo* ESCRT-III structures.

Recent observations, however, have challenged the dome model. [1] ESCRT-III complexes have also been observed to assemble into cone-like shapes, occasionally *in vitro* [18] and more often *in vivo*. [5, 19–21] Furthermore, *in vitro* experiments provide evidence that ESCRT-III spirals polymeryze most probably outwards, forming consecutively wider rings. [14] For these and other reasons, a new mechanism has been recently proposed as described by the ‘buckling model’, [1, 22] which proposes that the ESCRT-III complex assembles at the neck and grows away from the bud in the shape of a truncated cone, until further growth or assembly of specific ESCRT components triggers an instability that flattens the cone into a planar spiral, thereby leading to scission of the membrane neck, see Fig 1B. Because the main ingredient of the mechanism is not buckling, but rather the reverse of it, we will refer to it as the *flattening cone model* in the following.

A necessary ingredient of this newly proposed model is that flattening of the truncated cone will lead to narrowing, closure, and scission of the membrane neck. However, no quantitative, physical argument has been given so far as to why the membrane neck should become narrower and not wider as a consequence of flattening of the ESCRT-III complex. What are the minimal conditions necessary for membrane scission *via* flattening of the ESCRT-III cone? In particular, is there a minimal membrane–protein adhesion strength necessary for membrane scission, as was previously found for the dome model? [15] In the present study, we address and answer these questions. We show that flattening of the ESCRT-III cone can indeed lead to narrowing, closure, and scission of the membrane neck, as long as the membrane–protein adhesion strength is above a certain threshold value. This threshold value is around 0.18 mN/m.

In order to compare the flattening cone model with the dome model, we also reanalyzed the latter model. Our analysis shows that the original quantitative calculations for the dome model, presented in Ref [15], contained a computational error that led to incorrect conclusions from this model. In particular, we show that the neck closure transition is continuous rather than discontinuous as had been previously predicted. Our detailed comparison between the fission mechanisms based on dome-shaped and cone-shaped assemblies shows that the geometric constraints on the shape of the assembly required to induce scission are more stringent in the dome model than in the flattening cone model, and that the minimal membrane–protein adhesion strength necessary for membrane scission is many times lower in the flattening cone model compared to the dome model.

We also compute here for the first time the force exerted by the ESCRT-III complex onto the membrane neck, which squeezes the neck against itself and should be a direct measure of the scissioning capability of the complex. We will show that this constriction force varies from 60 to 100 pN in the dome model, and from 100 to 140 pN in the cone model.

We emphasize that, in this paper, we will focus on the final step of the process after a bud with an open neck has already formed, i.e. on how ESCRT-III drives the narrowing, closure, and ultimately the scission of this neck. In this way, we can evaluate and compare, in light of the available experimental data, [14, 15, 18] the dome [15] and flattening cone [1] mechanisms that have been proposed. Some of the earlier steps leading to ESCRT-induced bud formation have also been studied theoretically. The initial assembly of ESCRT components at the membrane may be associated with lipid sorting and membrane domain formation, which in turn can provide a driving force for initial budding of the membrane, both through the line tension of the domain boundary and through the spontaneous curvature of the membrane domain. [23] In particular, the assembly of ESCRT-I and II has been proposed to drive bud formation through the induction of negative Gaussian curvature of the membrane, [24, 25] through the generation of lipid domains with a strong line tension at the domain boundary, [24] and through a significant spontaneous curvature arising from the protein coat. [8] Budding of the membrane may also be driven by the buckling of the ESCRT-III Snf7 spiral, [14, 26] or by the cargo itself, as in the case of exocytic engulfment of viral capsids or nanoparticles. [1, 27] Moreover, we note that although we will use the word ‘bud’ for conciseness throughout the paper, the results described here can be applied not only to the fission of small buds, but also to membrane fission during cytokinesis. Indeed, cytokinetic abscission effectively corresponds to fission of a large, micron-sized bud within our description.

Similarly, we will focus only on the response of the membrane to the growth and deformation of the ESCRT assembly, assuming that this assembly either (i) grows in the shape of a dome, or (ii) grows in the shape of a cone which then flattens. It is beyond the scope of our study to elucidate how ESCRT may assemble into these specific shapes at the membrane, or what is the driving force for flattening in the case of the cone model. These dynamic processes are presumably governed by the energetics of association and deformation of the different ESCRT components and Vps4. While the elasticity of Snf7 spirals is now fairly well understood through *in vitro* experiments [14] and theory, [14, 26] it is still unclear how the remaining components interact with each other and with Snf7 in the full *in vivo* ESCRT assembly. In particular, it is possible that the ATP-dependent activity of Vps4 plays an important role in the growth and deformation of the assembly. [11, 12] For these reasons, a full model of ESCRT assembly at the membrane is still out of reach, and we instead focus on understanding and comparing the deformations of the membrane in response to two *postulated* mechanisms [1] of the ESCRT assembly.

The paper is organized as follows: First, we introduce the curvature-elastic theory used to compute the deformation energy of the membrane, the calculations for the dome and the flattening cone models, as well as the implementation of neck scission in the theory. We then describe the main results for the dome and flattening cone models. A discussion of the differences between our results and the incorrect results for the dome model previously reported in Ref [15] follows. Finally, we compare the dome and flattening cone models, and compute the adhesion-induced constriction forces exerted onto the membrane neck in both models.

## Methods

### Curvature elasticity and membrane-protein adhesion

We will describe the membrane by the theory of curvature elasticity, [28, 29] with the bending free energy density given by $F_{\text{be}}=2\kappa {\left(M-m\right)}^{2}$, where *M* is the (local) mean curvature of the membrane, *m* is the spontaneous curvature, and *κ* is the bending rigidity. A typical value of *κ*, which will be used throughout the paper, is *κ* = 20*k*_B_*T* ≃ 0.822 × 10^−19^ J at room temperature *T* = 25°C. For simplicity, we will focus on the case of symmetric bilayer membranes, with zero spontaneous curvature *m* = 0. In practice, our results will be applicable to weakly-asymmetric membranes as long as the corresponding spontaneous curvature *m* is much smaller than the inverse radius of the bud, with |*m*| ≪ 1/*R*_bu_. Some consequences of larger spontaneous curvatures will also be discussed throughout the paper. Furthermore, we will focus on the process of neck closure that represents a necessary step prior to membrane scission, but we will not explicitly consider the scission process itself. For this reason, we will not consider changes in the topology of the membrane and will omit the contribution of Gaussian curvature to the curvature energy, which is constant in the absence of topological changes. [29]

The adhesion between the membrane and the dome-like or cone-like ESCRT complex will be taken into account using the adhesion energy per unit area, *W* < 0, of the contact area between membrane and protein. [30] The *adhesive strength* |*W*| can contain, in general, contributions from generic interactions such as electrostatic or van der Waals forces, as well as from specific membrane–protein interactions.

**Fig 2:**
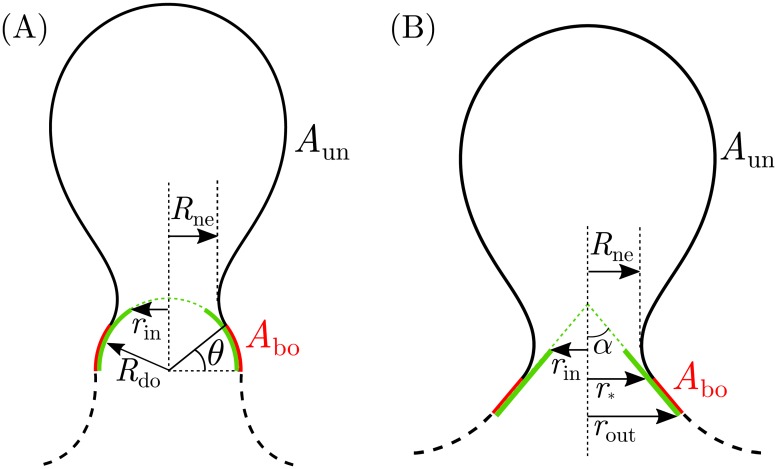
Geometry of dome-shaped and cone-shaped membrane–protein complexes. (A) The dome model, with dome radius *R*_do_ and inner radius *r*_in_; and (B) the truncated cone model, with cone apex angle *α*, inner radius *r*_in_ and outer radius *r*_out_. Both geometries are axisymmetric. The protein complex is represented by the solid green lines, while the dashed green lines are auxiliary lines representing the underlying spherical or conical shape of the complex. The unbound part of the membrane corresponding to the nascent bud (solid black segment, with area *A*_un_) with neck radius *R*_ne_ meets the part of the membrane bound to the protein (red segment, with area *A*_bo_) along the contact line. The position of the contact line is described (A) by the attachment angle *θ* in the dome model; and (B) by the attachment radius r_*_ in the cone model. The unbound part of the membrane away from the bud (dashed black segment) detaches from the protein (A) at the equator of the hemispherical dome in the dome model; and (B) at the outer radius *r*_out_ in the cone model. For low membrane tension, the shape of this unbound segment is catenoidal and does not contribute to the free energy of the system.

The membrane can be divided into three distinct segments: the unbound part of the membrane corresponding to the nascent bud (solid black segment in Fig 2), the part of the membrane bound to the protein in the proximity of the bud’s neck (red segment in Fig 2), and the remaining unbound part of the membrane away from the bud (dashed black segment in Fig 2). If the tension of the membrane is small enough, the unbound part of the membrane away from the bud will form a catenoidal shape with zero mean curvature and zero bending energy. [31] The membrane tension *Σ* can be considered small enough for our purposes as long as the radius of the membrane neck *R*_ne_ is several times smaller than the characteristic length scale $\xi \equiv \sqrt{\Sigma /\kappa }$. The typical tension of cellular membranes can range from 0.003 mN/m for epithelial cells to 0.3 mN/m for keratocytes. [32] Using the bending rigidity *κ* = 20*k*_B_*T*, we find that the length scale *ξ* varies from 16 nm for keratocytes to 165 nm for epithelial cells. Because we will consider only the process of neck closure with neck radii in the range of 3 to 25 nm, we can safely ignore the contributions of membrane tension in most cases, and the unbound part of the membrane away from the bud will be ignored in the following. The contributions of the two other membrane segments are non-zero, and are described below for the two different cases of dome-like and cone-like ESCRT complexes.

### Dome model

The part of the membrane bound to the dome-like ESCRT complex has a well defined shape imposed by the complex. For a hemispherical dome with radius *R*_do_, the combined bending and adhesion free energies of the bound membrane segment are given by

$$\begin{array}{c} F_{\text{bo}}\left(\theta \right)=(2\kappa -\left|W\right|R_{\text{do}}^{2})2\pi \sin\theta \end{array}$$ (1)

with the membrane attachment angle *θ* defined in Fig 2A. The attachment angle necessarily satisfies *R*_do_ cos *θ* ≥ *r*_in_, where *r*_in_ is the inner radius of the dome, which decreases continuously from *r*_in_ = *R*_do_ towards zero as the dome-like assembly grows. Following the experimental estimates in Ref [15], we will fix the dome radius to *R*_do_ = 25 nm.

The free energy of the unbound part of the membrane corresponding to the nascent bud is equal to $F_{\text{un}}=2\kappa \int_{A_{\text{un}}}dA\;M^{2}$, where *A*_un_ is the unbound area that forms the bud. The radius *R*_bu_ of the final, fully-formed bud is imposed by the condition that, at the initial stage of attachment with *θ* = 0°, the unbound area *A*_un_ be equal to $A_{0}=4\pi R_{\text{bu}}^{2}+2\pi R_{\text{do}}^{2}$ where the second term represents the membrane area that will be bound to the hemispherical dome after scission, see the last cartoon in Fig 1A. Both *R*_bu_ and *A*_0_ are therefore equivalent measures for the size of the bud. We note that fixing *A*_0_ provides only one possible definition of the bud radius: for example, one could alternatively fix *A*_un_, or fix the curvature of the membrane at the north pole of the unbound segment, as will be done in the case of the cone model, for which *A*_0_ is not well-defined. In practice, the choice of the constraint does not make any significant difference on the final results, which depend only on the radius of the bud at neck closure. The shape of the unbound membrane segment that minimizes the bending energy *F*_un_ for given bud radius *R*_bu_, while satisfying boundary conditions that ensure a smooth matching with the bound part of the membrane is then numerically calculated *via* the usual shooting method, [29, 33] for a range of values of the attachment angle *θ*. In this way, we can calculate the energy of the unbound segment *F*_un_(*θ*), and obtain the total free energy landscape of the system

$$\begin{array}{c} F_{\text{tot}}\left(\theta \right)=F_{\text{bo}}\left(\theta \right)+F_{\text{un}}\left(\theta \right) \end{array}$$ (2)

for given adhesive strength |*W*| and bud radius *R*_bu_ or initial bud area *A*_0_.

Alternatively, it is also possible to directly obtain the equilibrium states of such an energy landscape, i.e. the states for which ∂*F*_tot_(*θ*)/∂*θ* = 0, without having to calculate the full energy landscape, by considering the balance of forces at the contact line between the unbound and bound segments of the membrane. [30, 34] In this case, the attachment angle *θ* is not directly fixed in the shooting method, and is instead chosen so that the principal curvature perpendicular to the contact line *C*_⊥_ of the unbound segment satisfies the force balance condition $C_{⊥}=1/R_{\text{do}}-\sqrt{2|W|/\kappa }$ at the contact line. [30, 34]

### Flattening cone model

As in the case of the dome model, the shape of the membrane segment bound to the cone-like ESCRT complex is well defined. The combined bending and adhesion free energy of a membrane bound to a cone has the form

$$\begin{array}{c} F_{\text{bo}}\left(r_{*},r_{\text{out}}\right)=\pi \kappa \frac{\cos^{2}\alpha }{\sin\alpha }\log\frac{r_{\text{out}}}{r_{*}}-\pi \left|W\right|\frac{r_{\text{out}}^{2}-r_{*}^{2}}{\sin\alpha } \end{array}$$ (3)

with the apex angle *α* as well as the attachment radius r_*_ and the outer radius *r*_out_ defined in Fig 2B. The attachment radius necessarily satisfies *r*_in_ ≤ r_*_ ≤ *r*_out_, where *r*_in_ is the inner radius of the cone.

Again, the only free energy contribution of the unbound membrane segment corresponding to the nascent bud is provided by the bending energy $F_{\text{un}}=2\kappa \int_{A_{\text{un}}}dA\;M^{2}$, where *A*_un_ is the unbound area that forms the bud. The bud radius *R*_bu_ is imposed on the shape of the unbound membrane segment by the boundary condition at the north pole of this segment. Thus, we calculate the shape of the unbound segment under the condition that its mean curvature is equal to 1/*R*_bu_ at the north pole. For given values of the cone apex angle *α* and the bud radius *R*_bu_, the shape of the unbound membrane segment that minimizes the energy *F*_un_ is again calculated using the shooting method for a range of values of the attachment radius r_*_, from which we obtain the energy of the unbound segment *F*_un_(r_*_). The total free energy landscape of the system is then given by

$$\begin{array}{c} F_{\text{tot}}\left(r_{*},r_{\text{out}}\right)=F_{\text{bo}}\left(r_{*},r_{\text{out}}\right)+F_{\text{un}}\left(r_{*}\right). \end{array}$$ (4)

As emphasized in the *Introduction*, in the present work we elucidate the response of the membrane to the different shapes of the ESCRT complex. It is not our intention to discuss possible mechanisms for the formation of these shapes, or to characterize the driving force for cone flattening: a molecularly detailed theory of the assembly and elasticity of the different ESCRT components would be needed for this purpose. As a consequence, we do not know how the inner and outer radii *r*_in_ and *r*_out_ of the cone evolve with the apex angle *α* as the cone grows and flattens. This lack of knowledge does not present a problem because, as long as *r*_in_ ≤ r_*_, our results are independent of the precise value of *r*_in_. Moreover, the value of the outer radius *r*_out_ is irrelevant to the study of neck closure in the model, because (i) neck closure is dictated by the shape of the unbound membrane segment, which only depends on r_*_; and (ii) when calculating the equilibrium value of r_*_, by taking ∂*F*_tot_(r_*_, *r*_out_)/∂r_*_ = 0, we find that *r*_out_ drops out, that is, the value of r_*_ that minimizes the energy of the system is independent of the value that *r*_out_ might take. This independence applies as long as *r*_out_ ≥ r_*_: otherwise, the complex will not bind to the membrane.

As for the dome model, by considering the force balance at the contact line, it is possible to sidestep the calculation of the full energy landscape, and to directly obtain the equilibrium states for which ∂*F*_tot_(r_*_, *r*_out_)/∂r_*_ = 0. In this case, the attachment radius r_*_ is not directly fixed in the shooting method, and is instead chosen so that the principal curvature perpendicular to the contact line *C*_⊥_ of the unbound segment satisfies the force balance condition $C_{⊥}=-\sqrt{2|W|/\kappa }$ at the contact line. [30, 34]

### Neck closure and scission

The curvature-elastic theory used here is a coarse-grained theory that does not take the finite thickness of the membrane into account. As a consequence, the theory can describe membrane shapes with infinitely narrow necks (as described, e.g., in Ref [33]), and does not provide an explicit mechanism for the scission of the neck. Following Ref [15], we will assume that scission occurs when the neck radius *R*_ne_ approaches the typical thickness of a lipid membrane: we will take *R*_ne_ = 3 nm as the condition for membrane scission throughout the paper. We note, however, that our results are not very sensitive to the choice of the neck radius at which fission occurs, as long as this neck radius is several times smaller than the bud radius (and the dome radius in the case of the dome model).

As described in Ref [15] and confirmed by our own numerical calculations, the dome model with a dome radius *R*_do_ = 25 nm leads to a neck radius of 3 nm when the attachment angle is *θ* ≃ 75°, a value that depends only weakly on the size of the bud. This numerical observation can be understood using an analytical result for the scaling of narrow necks with the attachment angle, which was obtained in Ref [33] and has the form

$$\begin{array}{c} R_{\text{ne}}\approx R_{\text{do}}(1+\frac{R_{\text{do}}}{R_{\text{bu}}})\cos^{2}\theta \;\;\;\text{for}\;R_{\text{ne}}\ll R_{\text{do}}\text{,} \end{array}$$ (5)

see Eq 36 in Ref [33], with the particle radius *R*_pa_ replaced by the dome radius *R*_do_ and the attachment angle *θ* related to the wrapping angle *ϕ* by *θ* = *ϕ* − *π*/2 as well as with the spontaneous curvature *m* set equal to zero. For buds with total area *A*_0_ ranging from $25R_{\text{do}}^{2}$ to $180R_{\text{do}}^{2}$, numerical calculations show that the attachment angle required to achieve *R*_ne_ = 3 nm ranges from *θ* = 73.9° to *θ* = 72.1°. [15] Using the asymptotic equality in Eq 5, we predict *θ* ranging from 75.1° to 72.2°, in good agreement with the numerical results. Therefore, we will use *R*_ne_ = 3 nm and *θ* = 75° as equivalent conditions for membrane scission in the dome model.

## Results

### Dome-shaped assemblies of ESCRT proteins

**Fig 3:**
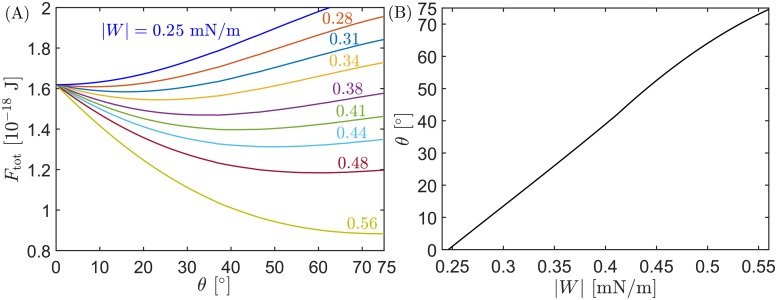
Energy landscape for dome-shaped assemblies. (A) Total energy of the system as a function of the attachment angle *θ*, for different values of the membrane–protein adhesion strength |*W*|. For |*W*| = |*W*|_as_ ≃ 0.25 mN/m, the energy landscape has a minimum at *θ* = 0°. As |*W*| increases beyond this value, the attachment angle *θ* of the minimum energy state (i.e. the optimal attachment angle) moves continuously towards higher *θ*-values, reaching *θ* = 75° (corresponding to neck closure and scission) for |*W*| = |*W*|_sc_ ≃ 0.56 mN/m. (B) Optimal attachment angle *θ* as a function of |*W*|. In both (A) and (B), the total area of the bud is $A_{0}=42R_{\text{do}}^{2}=26250$ nm^2^, corresponding to a final bud radius *R*_bu_ ≃ 42 nm.

Using the quantitative description of the dome model introduced in *Methods*, we can explore the free energy landscape *F*_tot_(*θ*) for neck closure in this model. In Fig 3A, we plot this energy landscape for several values of the membrane–protein adhesion strength |*W*|, for a fixed value of the bud area *A*_0_ = 42*R*_do_, which corresponds to a final bud radius *R*_bu_ ≃ 42 nm. For low |*W*|, with |*W*| < 0.25 mN/m, the energy landscape increases monotonically and does not have a minimum, implying that attachment of the membrane to the hemispherical dome is not energetically favorable, and therefore that assembly of the dome at the neck of the bud is impossible. For |*W*| = |*W*|_as_ ≃ 0.25 mN/m, the energy landscape develops a minimum at *θ* = 0°, which defines the first critical value of |*W*| above which assembly of the dome becomes possible. As |*W*| is increased further, the location of the energy minimum moves towards higher values of *θ*, i.e. the optimal attachment angle increases with increasing |*W*|. Finally, for |*W*| = |*W*|_sc_ ≃ 0.56 mN/m the optimal attachment angle becomes *θ* = 75°, which defines the second critical value |*W*|_sc_ for full closure and scission of the neck. The evolution of the optimal attachment angle with increasing |*W*| is shown in Fig 3B.

**Fig 4:**
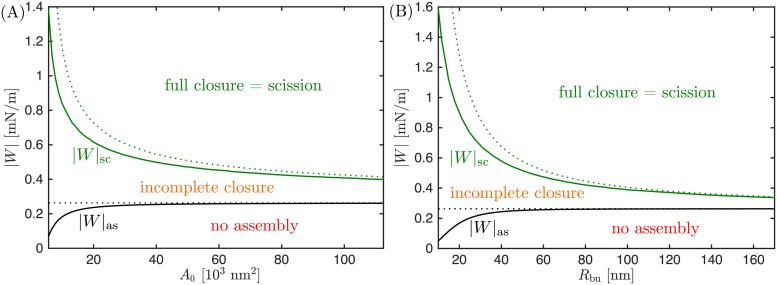
Dome-shaped assemblies as a function of bud size and adhesive strength |*W*|. The radius of the membrane neck will become sufficiently small (*R*_ne_ = 3 nm) and thus full neck closure and scission will occur only if the membrane–protein adhesion strength |*W*| is sufficiently large with |*W*| > |*W*|_sc_; the closure of the neck will be incomplete for intermediate values of |*W*| with |*W*|_sc_ > |*W*| > |*W*|_as_; and the dome will not assemble at all if the adhesive strength |*W*| is too small with |*W*| < |*W*|_as_. The critical values of the adhesive strength |*W*|_as_ corresponding to *θ* = 0° and |*W*|_sc_ corresponding to *θ* = 75° are plotted (A) as a function of the total bud area $A_{0}=4\pi R_{\text{bu}}^{2}+2\pi R_{\text{do}}^{2}$; and (B) equivalently as a function of the final bud radius *R*_bu_. The solid lines correspond to numerical results, whereas the dotted lines correspond to the analytical approximations given by Eqs 7 and 9.

The two critical values of |*W*| corresponding to assembly of the dome (optimal attachment angle *θ* = 0°) and membrane scission (*θ* = 75°) should depend on the size of the bud. We have plotted these two critical values in Fig 4: In (a), the size of the bud is measured by total bud area *A*_0_, while in (b) the size of the bud is measured *via* the radius *R*_bu_ of the resulting fully-formed bud. As explained in *Methods*, both measures of bud size are related by $A_{0}=4\pi R_{\text{bu}}^{2}+2\pi R_{\text{do}}^{2}$, where *R*_do_ = 25 nm is the radius of the protein dome. We find that the critical value |*W*|_as_, corresponding to *θ* = 0°, increases with increasing bud size; whereas the second critical value of |*W*|_sc_, corresponding to *θ* = 75°, decreases with increasing bud size. As a consequence, for small bud sizes, there is a wide range of |*W*|-values for which the dome can assemble at the neck but neck closure and scission are not achieved, a situation we call “incomplete closure” (in Ref [33], we used the terminology “closed and open neck” instead of “complete and incomplete closure”). As the buds grow larger and larger, the two critical values of |*W*| approach each other, and the region of incomplete closure becomes narrower and narrower. For buds that are much larger than the dome radius, the two critical values converge and we would find a direct transition from no assembly of the dome to full closure and scission of the neck as |*W*| is increased, without a region of incomplete closure in between.

It is interesting to note that the geometry of the membrane in the dome model is identical to that of exocytic engulfment of spherical particles by vesicles, i.e. of the engulfment of a particle that originates from the inside compartment of a vesicle by the vesicle membrane. [27] For this reason, results that have been obtained for particle engulfment can be directly applied to the study of the dome model. In particular, in Ref [35], we derived an analytical expression for the optimal attachment angle valid in the limit of particles much smaller than the vesicle, which in the case of the dome model corresponds to the limit of buds much larger than the dome radius, see Eq 33 in Ref [35]. The relationship between the notation used in Ref [35] and the one used here is as follows. The parameter *q* is related to *θ* here *via* *q* = (1 + sin *θ*)/2, the mean curvature *M* of the membrane corresponds to 1/*R*_bu_, and the spontaneous curvature is *m* = 0. In addition, the particle radius *R*_pa_ corresponds to the radius *R*_do_ of the dome, so that the contact mean curvature is $M_{\text{co}}=-\sqrt{|W|/2\kappa }+1/R_{\text{do}}$. Using these notational relationships in Eq 33 of Ref [35], we obtain the asymptotic equality

$$\begin{array}{c} \left|W\right|\approx 2\kappa {\left(\frac{1}{R_{\text{do}}}+\frac{\sin\theta }{R_{\text{bu}}}\right)}^{2} \end{array}$$ (6)

for large *R*_bu_/*R*_do_. For *θ* = 0°, the asymptotic equality in Eq 6 leads to the minimal value

$$\begin{array}{c} {\left|W\right|}_{\text{as}}\approx \frac{2\kappa }{R_{\text{do}}^{2}} \end{array}$$ (7)

of the adhesive strength required for dome assembly. Moreover, we can use the asymptotic equality in Eq 5 for the neck radius, which to lowest order in *R*_do_/*R*_bu_ is *R*_ne_ ≈ *R*_do_ cos^2^ *θ* or equivalently sin^2^ *θ* ≈ 1 − *R*_ne_/*R*_do_, in order to write Eq 6 as a function of the neck radius *R*_ne_ instead of the attachment angle *θ*. In this way, we obtain the relation

$$\begin{array}{c} \left|W\right|\approx 2\kappa {\left(\frac{1}{R_{\text{do}}}+\frac{1}{R_{\text{bu}}}\sqrt{1-\frac{R_{\text{ne}}}{R_{\text{do}}}}\right)}^{2}\;\;\;\text{for}\;R_{\text{bu}}\gg R_{\text{do}}\text{.} \end{array}$$ (8)

When we now use the specific values *R*_do_ = 25 nm and *R*_ne_ = 3 nm, we obtain the minimal value

$$\begin{array}{c} {\left|W\right|}_{\text{sc}}\approx 2\kappa {\left(\frac{1}{\text{25}\;\text{nm}}+\frac{0.938}{R_{\text{bu}}}\right)}^{2}\;\;\;\text{for}\;R_{\text{bu}}\gg 25\;\text{nm} \end{array}$$ (9)

of the adhesive strength required for closure and scission of the membrane neck as a function of the bud radius *R*_bu_. Eqs 7 and 9 are plotted as the dotted lines in Fig 4, using *κ* = 0.822 × 10^−19^ J. Inspection of Fig 4 shows that the dotted lines provide a very good approximation to the solid lines as obtained numerically for large buds corresponding to large *A*_0_ and a reasonable approximation for small buds with small *A*_0_. The first critical value, |*W*|_as_, corresponding to the onset of dome assembly with *θ* = 0° is predicted to be independent of the bud size, which represents a good approximation except for very small bud sizes. The second critical value |*W*|_sc_ corresponding to neck closure and scission does depend on bud size but, as observed numerically, approaches the first critical value in the limit of very large buds with *R*_bu_ ≫ *R*_do_.

The results above were derived for a symmetric bilayer with zero spontaneous curvature *m* = 0. However, we can again use the known results for exocytic engulfment of particles by vesicles [27, 35] in order to understand the effects that a non-zero membrane spontaneous curvature will have on neck closure and scission by dome-shaped assemblies. For negative or small positive spontaneous curvatures with *m* < 1/*R*_bu_, the neck closure transition is still continuous, and the behaviour of the system is qualitatively similar to the case with *m* = 0. The two critical values of the adhesive strength |*W*|_as_ and |*W*|_sc_ both decrease with increasing spontaneous curvature, and at the same time approach each other until they meet for *m* ≈ 1/*R*_bu_, in which case we have |*W*|_as_ ≈ |*W*|_sc_.

### Cone-shaped assemblies of ESCRT proteins

**Fig 5:**
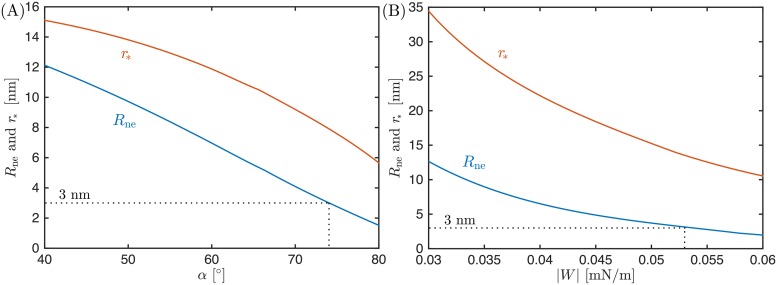
Evolution of the neck radius *R*_ne_ and the attachment radius r_*_ for cone-shaped assemblies. (A) As a function of the cone apex angle *α* for fixed membrane–protein adhesion strength |*W*| = 0.2 mN/m; and (B) as a function of the adhesive strength |*W*| for fixed cone apex angle *α* = 90°, i.e. for a fully flattened cone. The neck will close and scission will occur when the neck radius reaches *R*_ne_ = 3 nm, indicated by the dotted line. In both (A) and (B), the bud radius is *R*_bu_ = 42 nm.

We used the numerical procedure outlined in *Methods* to determine the shape of buds in the cone model, for many different values of the bud radius *R*_bu_, membrane–protein adhesion strength |*W*|, and cone apex angle *α*. In general, we find that both an increase in |*W*| at constant *α*, and more importantly an increase in *α* at constant |*W*| (i.e. flattening of the cone) both lead to narrower and narrower necks and eventually to membrane scission when the neck radius reaches *R*_ne_ = 3 nm. As an example, in Fig 5, we plot the neck radius *R*_ne_ and the attachment radius r_*_ as a function of (a) the apex angle *α* for fixed |*W*| = 0.2 mN/m, and (b) the adhesive strength |*W*| for a fully-flattened cone with fixed *α* = 90°. In both cases, the bud radius is fixed to *R*_bu_ = 42 nm.

**Fig 6:**
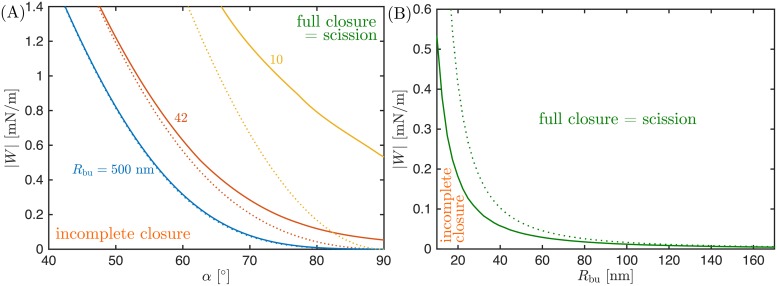
Regimes of incomplete and full closure for cone-shaped assemblies. (A) Critical value |*W*|_sc_ of the adhesive strength |*W*| for which the neck radius becomes *R*_ne_ = 3 nm, leading to neck closure and scission, as a function of the cone apex angle *α*, for bud radii *R*_bu_ = 500, 42 and 10 nm. For fixed |*W*|, flattening of the cone (i.e. increasing *α* towards 90°) leads to scission, except for low |*W*| for which not even full flattening of the cone (*α* = 90°) can achieve scission. This minimal value of the adhesive strength necessary for scission at full flattening is shown in (B), as a function of the bud radius *R*_bu_. In both (a) and (b), solid lines correspond to numerical results, while dotted lines correspond to the analytical approximations given by Eq 12 in (A) and by Eq 13 in (B).

It was first speculated in Ref [22] that flattening of the ESCRT cone could lead to narrowing and eventually scission of the membrane neck, and this hypothesis was described in more detail in Ref [1]. However, no quantitative explanation of why cone flattening should lead to narrowing of the neck has been given so far. That cone flattening can indeed induce neck closure is an important result of the present study. In Fig 6A we calculate the minimal apex angle *α* required for neck closure closure as a function of the adhesive strength |*W*| of the membrane-protein interaction. We find that, above a certain critical adhesive strength, flattening of the cone always leads to neck closure. This critical adhesive strength, which increases with decreasing bud size as shown in Fig 6B, corresponds to the case when neck closure occurs precisely at full flattening of the cone with apex angle *α* = 90°. For adhesive strengths above this critical value, neck closure can occur even before the cone is completely flat, i.e., with *α* < 90°.

**Fig 7:**
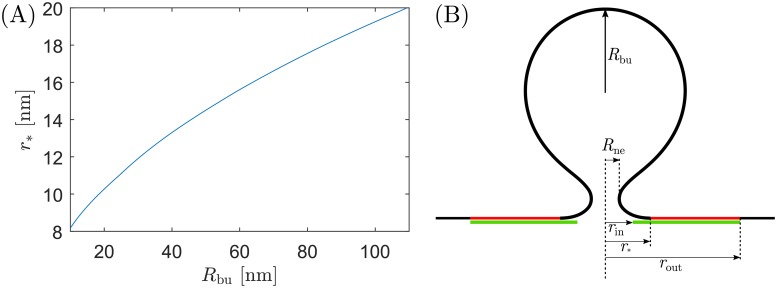
Geometric constraints on fission by cone-shaped assemblies. (A) Attachment radius r_*_ at the moment of neck closure corresponding to *R*_ne_ = 3 nm, as a function of bud radius *R*_bu_, for the particular case of a fully flattened cone with apex angle *α* = 90°. For scission to be possible in this model, the inner and outer radii of the cone must satisfy *r*_in_ ≤ r_*_ ≤ *r*_out_, see (B).

We note that, in contrast to the dome model, we cannot calculate an explicit value of the adhesive strength |*W*|_as_ below which assembly of the cone is impossible. Such a value would correspond to the value for which the attachment radius r_*_ is equal to the outer radius *r*_out_ of the cone but, in the absence of a detailed theory for the remodelling of the cone, we do not know how *r*_out_ evolves with varying apex angle *α*. The theory presented here is therefore valid only as long as, at neck closure (i.e. when *R*_ne_ = 3 nm), the inner and outer radii of the protein cone satisfy *r*_in_ ≤ r_*_ ≤ *r*_out_, see Fig 2B. In Fig 7, we plot the value of r_*_ at the moment of neck closure as a function of bud radius, for the particular case of a fully flattened cone with *α* = 90°.

Once again, an analogy can be drawn between the geometry of the membrane in the cone model and the one of exocytic engulfment of spherical particles by vesicles. Indeed, the shape of the unbound segment of the membrane in the cone model, with cone apex angle *α* and attachment radius r_*_, should be equivalent to that of the dome model with an effective “attachment angle” *θ*_eff_ = *α*, and an effective “dome radius” *R*_do,eff_ = r_*_/ cos *α*. Furthermore, as explained above, there is a direct correspondence between the dome model and exocytic engulfment of spherical particles by vesicles. In Ref [35], we obtained an approximate expression for the free energy of the unbound segment of the membrane valid in the limit of small particles, which in the cone model would correspond to *R*_bu_ ≫ r_*_/ cos *α*. In the context of the cone model, this expression becomes

$$\begin{array}{c} F_{\text{un}}\approx 8\pi \kappa -4\pi \kappa \frac{r_{*}}{R_{\text{bu}}}\cos\alpha . \end{array}$$ (10)

This energy can be added to the free energy of the bound segment *F*_bo_ in Eq 3 in order to obtain an analytical expression for the total free energy *F*_tot_(r_*_, *r*_out_) = *F*_bo_(r_*_, *r*_out_) + *F*_un_(r_*_). Using this latter expression, we can now obtain an explicit condition for the equilibrium of the system as described by ∂*F*_tot_(r_*_, *r*_out_)/∂r_*_ = 0, which leads to

$$\begin{array}{c} \left|W\right|\approx \frac{\kappa \cos^{2}\alpha }{2r_{*}^{2}}+\frac{2\kappa \cos\alpha \sin\alpha }{r_{*}R_{\text{bu}}}. \end{array}$$ (11)

This equation provides the adhesive strength which is required to obtain a given equilibrium value of r_*_. In order to obtain the minimal value of the adhesive strength |*W*|_sc_ that leads to membrane scission, however, we would like to express Eq 11 as a function of the neck radius *R*_ne_ instead of r_*_. To achieve this change of variables, we can use the scaling relation for the neck radius in Eq 5, which in the case of the cone model (i.e. inserting the “effective” values described above) becomes to lowest order *R*_ne_ ≈ r_*_ cos *α* or r_*_ ≈ *R*_ne_/ cos *α*. Substituting this asymptotic equality into Eq 11, we finally obtain

$$\begin{array}{c} {\left|W\right|}_{\text{sc}}\approx \frac{\kappa \cos^{4}\alpha }{2R_{\text{ne}}^{2}}+\frac{2\kappa \cos^{2}\alpha \sin\alpha }{R_{\text{ne}}R_{\text{bu}}}\;\;\;\text{with}\;R_{\text{ne}}=3\;\text{nm} \end{array}$$ (12)

which is plotted as the dotted lines in Fig 6A. We see that Eq 12 provides a good approximation to the numerical results, as long as the radius of the bud is sufficiently large.

The approximation in Eq 12 has one caveat: it completely breaks down for a fully flattened cone, i.e. for *α* = 90°, in which case it always predicts |*W*|_sc_ = 0 independently of the bud size, which clearly does not agree with the numerical results. This failure of the approximation reflects the fact that its derivation was only valid for *R*_bu_ ≫ r_*_/ cos *α*: the latter condition becomes impossible to satisfy when *α* approaches 90°, because r_*_/ cos *α* increases towards infinity. A different approach must be used to approximate the required value of |*W*| for neck closure at a fully flattened cone. In Ref [33], we obtained an exact condition for the closure of a membrane neck connecting a bud to a membrane that is adhering to a planar substrate. In the notation of the present work, this condition has the form

$$\begin{array}{c} {\left|W\right|}_{\text{sc}}\approx \frac{2\kappa }{R_{\text{bu}}^{2}}\;\;\;\text{for}\;\alpha =90{}^{\circ}\text{.} \end{array}$$ (13)

Because this condition corresponds to the formation of an infinitesimally narrow neck, it should become a good approximation to the numerical results for sufficiently large buds, as confirmed by Fig 6B, where Eq 13 corresponds to the dotted line.

For membranes with non-zero spontaneous curvature *m* ≠ 0, we expect from the known results for budding of a supported lipid bilayer [33] that the minimal adhesive strength required for scission |*W*|_sc_ will decrease with increasing spontaneous curvature. In particular, the value of |*W*|_sc_ for a fully flattened cone with *α* = 90° will have the form |*W*|_sc_ ≈ 2*κ*(1/*R*_bu_ − 2*m*)^2^ for sufficiently large buds.

## Discussion

### Dome model: Comparison with the results of Fabrikant *et al*

The quantitative dome model as introduced in *Methods* was already explored in Ref [15]. Unfortunately, the authors miscalculated the free energy of the bound membrane segment, our Eq 1, obtaining an incorrect (1 − cos *θ*) factor instead of the correct factor sin *θ*, see Eq 2 in Ref [15]. As a consequence, their predictions were incorrect both qualitatively as well as quantitatively, as described in the following.

Our Figs 3A, 3B and 4A can be directly compared to Figs 4, 5B, and 5C in Ref [15], respectively, revealing many important differences. One important difference is that, in Ref [15], the neck closure was predicted to be *discontinuous*: for low |*W*|, the system has a single stable state with *θ* ≃ 0°; when |*W*| increases beyond a certain value the system exhibits a coexistence of two (meta)stable states, one with *θ* ≃ 0° and the other one with *θ* = 75°; finally, when |*W*| becomes sufficiently large, the state with *θ* ≃ 0° becomes unstable and the system undergoes a discontinuous transition towards neck closure and scission with *θ* = 75°. However, using the correct equations, we showed here that the neck closure proceeds in a *continuous* manner: as |*W*| is increased, the attachment angle *θ* increases continuously from *θ* = 0° to *θ* = 75°, including all angles in between. Therefore, the dome model does not predict the coexistence between two states or a discontinuous change in the attachment angle *θ*.

One may wonder if, beyond being interesting from a physics perspective, the distinction between a discontinuous or continuous neck closure transition can have any biological significance. In principle we expect that, as long as the ESCRT pathway functions properly, the protein–membrane adhesive strength |*W*| will be larger than the minimum value required for scission, |*W*|_sc_. In this case, whether the transition is continuous or discontinuous is not important, because in both cases the membrane will bind to all the available dome surface. However, if the ESCRT pathway is malfunctioning or intentionally perturbed so that the adhesion strength |*W*| is lower than |*W*|_sc_, the discontinuous transition as obtained in Ref [15] would erroneously predict no closure of the neck at all (*θ* ≈ 0°), whereas the continuous transition obtained here would predict the existence of what we call “incomplete closure” in Fig 4, i.e. cases in which the membrane adheres only partially to the dome (0° < *θ* < 75°).

Another important difference applies to the initial assembly of the dome at the neck. In Ref [15], it was found that even for arbitrarily low adhesive strengths |*W*| there will always be an equilibrium attachment angle with *θ* ≳ 0°, implying that assembly of the dome at the neck is always energetically favorable. Using the correct equations, on the other hand, we have shown that below a critical value |*W*|_as_ of the adhesive strength |*W*|, an equilibrium attachment angle does not exist and therefore dome assembly at the neck is impossible in this case. This minimal adhesive strength necessary for assembly of the dome is roughly independent of the radius of the bud and behaves as ${\left|W\right|}_{\text{as}}\approx 2\kappa /R_{\text{do}}^{2}$ for intermediate and large bud sizes as follows from Eq 6 and illustrated in Fig 4B.

The main qualitative feature of the dome model, i.e. that neck closure and scission will occur if the adhesive strength |*W*| between the dome and the membrane is large enough, remains unchanged. Quantitatively, however, we find that the minimal value |*W*|_sc_ required for neck closure and scission is significantly larger than predicted in Ref [15]: In the latter study, the minimal values |*W*|_sc_ varied between 0.6 mN/m and 0.35 mN/m for buds with areas ranging from *A*_0_ = 5625 nm^2^ to 112500 nm^2^. In contrast, we predict minimal values between 1.4 mN/m and 0.4 mN/m for the same range of bud areas. Therefore, for small buds, the minimal membrane–protein adhesion strength |*W*|_sc_ required for membrane scission by dome-shaped ESCRT assemblies is over two-fold higher than previously predicted.

### Comparison of dome-shaped and cone-shaped ESCRT assemblies

The dome and the flattening cone models of membrane fission by ESCRT are obviously different in the putative behavior of the ESCRT assembly: in the dome model, the assembly is assumed to grow *towards* the bud once assembled at the neck, whereas in the cone model the assembly is assumed to grow *away* from the neck. [1] Furthermore, in the dome model the complex grows forming a hemispherical shape of fixed radius *R*_do_ ≃ 25 nm, [15] whereas in the cone model the complex forms a conical shape with flattens as it grows.

Our results imply that the response of the membrane to the growing ESCRT assembly is rather different in the two models. In the dome model, scission may occur as long as the membrane–protein adhesive strength is large enough, with |*W*| ≥ |*W*|_sc_, in which case the preferred, equilibrium attachment angle *θ* is close to or larger than 75°. As a consequence, as the dome grows, the membrane will closely follow the growth of the dome, and be pinned to the inner edge of this dome, as depicted in Fig 1A. In the cone model, on the other hand, the equilibrium attachment radius r_*_ depends on the apex angle of the cone, and becomes smaller as the cone flattens, see Fig 5A. In general, the inner radius *r*_in_ of the cone may be smaller than the attachment radius, with *r*_in_ ≤ r_*_, in which case the membrane will not be pinned to the inner edge of the cone as it grows and flattens. This process is depicted in Fig 1B. The membrane will however be pinned to the inner edge of the cone if the latter is wide enough, i.e. if the inner radius of the cone is larger than the equilibrium attachment radius, with *r*_in_ > r_*_.

The two assembly mechanisms also differ with respect to the upper bound that they impose on the inner radius of the ESCRT assembly *r*_in_, as defined in Fig 2. For the dome model, we have found, in accordance with Ref [15], that for neck closure and scission to be possible, the attachment angle of the membrane to the dome has to be at least *θ* ≃ 70° (for the fission of very large buds, as obtained from Eq 5 with *R*_do_ / *R*_bu_ ≪ 1). This implies that, for neck scission to be at all possible in the dome model with a dome radius *R*_do_ = 25 nm, the inner radius of the ESCRT assembly has to be smaller than *R*_do_ cos *θ* ≃ 9 nm, and in general should be even smaller for the fission of smaller buds to be possible. For the flattening cone model, on the other hand, we find that the same inner radius of *r*_in_ ≈ 9 nm would be sufficient to drive fission of buds as small as *R*_bu_ ≃ 20 nm, see Fig 7. Therefore, the constraints imposed upon the geometry of the ESCRT assembly by the dome model are more stringent than those imposed by the flattening cone model.

Finally, a key difference between cones and domes is the magnitude of the minimal value |*W*|_sc_ for the adhesive strength between the membrane and the protein complex that is required for membrane scission in each of the two models. This minimal value depends on the bud size, and is larger for smaller buds, see Fig 4B for the dome model and Fig 6B for the cone model. Comparing both figures, we find that the minimal adhesive strength required for membrane scission in the cone model is much smaller than in the dome model. As an example, for the scission of small buds with *R*_bu_ = 20 nm, the cone model requires a minimal adhesive strength of |*W*|_sc_ = 0.18 mN/m, whereas the dome model requires 0.90 mN/m, a five-fold higher value of the minimal adhesive strength. For larger buds with *R*_bu_ = 100 nm, the cone model requires a minimal adhesive strength of 0.012 mN/m, whereas the dome model requires 0.39 mN/m, higher by a factor of 30.

Some estimates for the adhesion strength |*W*| between the ESCRT complex and the membrane can be found in the literature. First, in Ref [15], measurements of the binding kinetics of CHMP2A (Vps2) and CHMP3 (Vps24) monomers to DOPS-SOPC membranes were used to estimate an adhesive strength |*W*|≃3.45 mN/m. Second, in Ref [14], tube pulling experiments were used to measure the polymerization energy per unit area, *μ*, of Snf7 on DOPC-DOPS membranes, obtaining a value *μ* ≃ 0.31 mN/m. Because the polymerization energy lumps together binding between monomers and adhesion to the membrane, this value can be taken to imply an *upper bound* for the adhesive strength between Snf7 and the membrane, i.e., |*W*|≤0.31 mN/m. Comparing these two estimates with the values of |*W*|_sc_ described in the previous paragraph, we find that the former estimate (for adhesion of Vps2-Vps24) would be sufficient to drive neck closure in both the dome and cone models, whereas the latter estimate (for adhesion of Snf7) would be sufficient for closure in the cone model, but not in the dome model. Moreover, as described below, a lower value of |*W*|_sc_ will lead to larger adhesion-induced constriction forces at the neck.

### Adhesion-induced constriction forces

**Fig 8:**
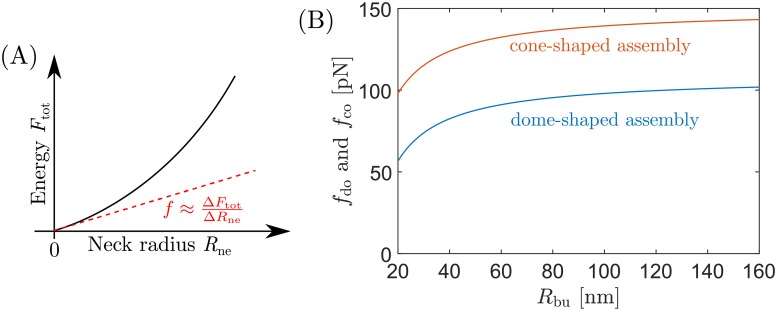
Adhesion-induced constriction forces exerted by the ESCRT complex onto the membrane neck. (A) The constriction force is given by $f={\partial F_{\text{tot}}/\partial R_{\text{ne}}|}_{R_{\text{ne}}=0}$, with the total free energy as in Eqs 2 and 4 for the dome and cone model, respectively. Thus, opening a closed neck by a small amount Δ*R*_ne_ implies the energetic cost of Δ*F*_tot_ ≈ *f*Δ*R*_ne_. (B) Force as a function of the radius *R*_bu_ of the bud, for a dome with radius *R*_do_ = 25 nm (blue line), and for a fully flattened cone (red line). In both cases, the membrane–protein adhesion strength is |*W*| = 3.45 mN/m. [15] The forces are given by Eqs 15 and 16 for the dome and the cone, respectively.

The large difference in the minimal adhesive strength |*W*|_sc_ required for membrane scission for dome-shaped and cone-shaped ESCRT assemblies could have important implications in their effectiveness to catalyze membrane fission. Indeed, we showed in Ref [33] that, when a membrane neck is closed by adhesion, there will be a constriction force squeezing the neck against itself if the adhesive strength |*W*| is larger than the minimal value |*W*|_sc_ required for neck closure. Furthermore, for |*W*| > |*W*|_sc_, the force increases monotonically with increasing |*W*|. The magnitude of the adhesion-induced constriction force exerted onto the neck should be directly related to the capability of the ESCRT complex to induce membrane fission. In Ref [33], an exact analytical expression for the force was derived in the limit of small neck radii *R*_ne_. The latter force is given by

$$\begin{array}{c} f=\frac{\partial F_{\text{tot}}}{\partial R_{\text{ne}}}{|}_{R_{\text{ne}}=0} \end{array}$$ (14)

with the total free energy given by Eqs 2 and 4 for the dome and the cone model, see Fig 8A, and is defined to be positive if it squeezes the neck against itself. For an adhesive hemispherical dome of radius *R*_do_, the adhesion-induced constriction force exerted onto the neck is

$$\begin{array}{c} f_{\text{do}}\approx 4\pi \kappa (\sqrt{\frac{\left|W\right|}{2\kappa }}-\frac{1}{R_{\text{do}}}-\frac{1}{R_{\text{bu}}}) \end{array}$$ (15)

whereas the adhesion-induced constriction force exerted by a fully flattened cone is

$$\begin{array}{c} f_{\text{co}}\approx 4\pi \kappa (\sqrt{\frac{\left|W\right|}{2\kappa }}-\frac{1}{R_{\text{bu}}}). \end{array}$$ (16)

We note that Eq 16 is recovered from Eq 15 for large *R*_do_, given that a large radius of curvature corresponds to a flat surface. In order to make the equations more instructive, we can rearrange the terms on the right hand side of each equation and rewrite the adhesion-induced constriction forces as

$$\begin{array}{c} f_{\text{do}}\approx 2\pi \sqrt{2\kappa }(\sqrt{\left|W\right|}-\sqrt{2\kappa {\left(\frac{1}{R_{\text{do}}}+\frac{1}{R_{\text{bu}}}\right)}^{2}}) \end{array}$$ (17)

and

$$\begin{array}{c} f_{\text{co}}\approx 2\pi \sqrt{2\kappa }(\sqrt{\left|W\right|}-\sqrt{\frac{2\kappa }{R_{\text{bu}}^{2}}}). \end{array}$$ (18)

Now, we can readily identify the second term inside the parenthesis of these equations as the square root of the minimal adhesive strength |*W*|_sc_ required for scission as derived above for domes and cones: in Eq 17, the term corresponds to Eq 9 with *R*_ne_ = 0, whereas in Eq 18 the term corresponds to Eq 13. In both cases, the fact that the neck radius is in reality not zero, but finite with *R*_ne_ ≃ 3 nm, introduces a small correction that acts to slightly increase the adhesion-induced force onto the neck.

We now use the upper estimate for the adhesive strength, |*W*|≃3.45 mN/m described in the previous section, in order to compare the constriction forces exerted by the ESCRT assembly in the dome and the cone model (for the lower estimate |*W*|≃0.31 mN/m, neck closure is impossible in the dome model). Using this value of |*W*|, together with *κ* = 20*k*_B_*T* and *R*_do_ = 25 nm, we have plotted the forces exerted at the neck as a function of bud radius in Fig 8B. We predict that the force exerted at the neck of a bud with radius *R*_bu_ = 20 nm will be *f*_do_ ≃ 57 pN in the dome model and *f*_co_ ≃ 98 pN in the cone model, i.e. 72% larger for the cone model. For a larger bud with radius *R*_bu_ = 100 nm, we find *f*_do_ ≃ 98 pN and *f*_co_ ≃ 139 pN, or 42% larger for the cone model. More generally, combining Eqs 15 and 16, we find that the force exerted onto the neck in the cone model is always larger than in the dome model, with a difference given by

$$\begin{array}{c} f_{\text{co}}-f_{\text{do}}\approx \frac{4\pi \kappa }{R_{\text{do}}} \end{array}$$ (19)

which, surprisingly, is independent of the bud size and of the membrane–protein adhesion strength. As a consequence, the force exerted at the membrane neck by a fully flattened cone will always be ≃41 pN larger than the force exerted by a dome with radius *R*_do_ = 25 nm.

As described in Ref [33], the presence of a non-zero membrane spontaneous curvature can also have an effect on the magnitude of the constriction forces at the neck. More precisely, the effect of a non-zero spontaneous curvature *m* ≠ 0 is to add a term 8*πκm* to the force, both for dome-shaped as well as for cone-shaped assemblies. Therefore, positive spontaneous curvatures act to increase the constriction force at the neck, whereas negative spontaneous curvatures act to decrease it.

### Conclusion

In summary, we have explored in quantitative detail the narrowing, closure, and scission of membrane necks within two previously proposed models for fission of membranes by ESCRT: the dome model and the flattening cone model (also known as the buckling model). [1] Concerning the dome model, we realized that a previous quantitative study of the model [15] contained a computational error which led to wrong predictions for this model, both qualitatively as well as quantitatively. We showed that the neck closure transition for dome-shaped assemblies, which had been incorrectly predicted to be discontinuous, is in fact continuous. Furthermore, the correct calculations show that the minimal membrane–protein adhesion strength required for membrane scission by dome-shaped assemblies is over two-fold higher than had been previously predicted. For cone-shaped assemblies, we have demonstrated within the theoretical framework of curvature elasticity and membrane-protein adhesion that cone flattening as recently proposed [1, 22] can indeed lead to closure and scission of the membrane neck. In fact, we showed that neck closure and scission are ‘easier’ in the cone model than in the dome model, in the sense that the minimal membrane–protein adhesion strength required for membrane scission is lower in the cone model. Furthermore, the upper bound imposed by both models on the inner radius of the ESCRT assembly is more restrictive in the dome model than in the flattening cone model. Finally, we have calculated for the first time the force exerted by the ESCRT complex on the membrane neck, in both models. The forces exerted at the neck are always larger in the cone model, with values ranging between 60 and 100 pN in the dome model, and between 100 and 140 pN in the cone model.

Further work, both experimental and theoretical, will be needed to elucidate the precise mechanism by which ESCRT participates in membrane fission. In particular, while the present work has focused on the deformations of the membrane in response to the growth of the ESCRT complex in dome-like or cone-like shapes, we have not attempted to describe the origin of these shapes, or the driving force behind the flattening of the cone in the case of the cone model. A molecularly detailed theory including the energetics and assembly dynamics of the different ESCRT components needs to be developed in order to achieve a full understanding of the process. Nevertheless, our study shows that cone flattening is an effective mechanism to induce membrane fission, and that in fact this mechanism achieves fission more efficiently than the assembly of a static dome.
